# Epidemiology of Traffic Fatalities among Motorcycle Users in East Azerbaijan, Iran

**DOI:** 10.1155/2018/6971904

**Published:** 2018-08-19

**Authors:** Homayoun Sadeghi-Bazargani, Bahram Samadirad, Hojjat Hosseinpour-Feizi

**Affiliations:** ^1^Road Traffic Injury Research Center, Tabriz University of Medical Sciences, Tabriz, Iran; ^2^Legal Medicine Research Center, Forensic Medicine Organization, Tehran, Iran; ^3^Department of Orthopedics, Tabriz University of Medical Sciences, Tabriz, Iran

## Abstract

**Background:**

The aim of this study was to determine some epidemiological aspects of motorcycle user traffic fatalities including the crash mechanisms and injury patterns in East Azerbaijan, Iran (2006-2016).

**Methods:**

A total of 1840 motorcycle user mortal cases registered in East Azerbaijan forensic medicine database, in Iran, were analyzed over the time period between March 2006 and March 2016. The distribution and associations of both victim- and crash-related variables such as crash mechanism, types of involved vehicles, types of injuries, and demographic characteristics were investigated. Data were analyzed by Stata v.13 statistical software package.

**Results:**

Of the 9435 RTI deaths, 1840 (19.5%) were motorcycle users of whom 96.5% were male (mean age of 32.3 ± 18.5 years). The majority of accident mechanisms were motorcycle-vehicle crashes (77.8%), followed by rollover (11.8%). Cars were the leading counterpart crash vehicle comprising about 34.8% of the motorcycle user mortalities. Inner city traffic injuries were the reason for 744 (40.7%) of the motorcycle user mortalities. Head trauma was the main cause of death. About 82% of the victims were motorcycle riders and the remainder were pillion passengers. A decreasing trend of fatal traffic accidents was observed over the study period for both the motorcycle user fatalities and other traffic injuries. The percentage of motorcycle mortalities over all traffic mortalities had a decreasing trend over the past nine years from March 2007 to March 2016 reaching a figure as low as 15.2% through the last year of study.

**Conclusions:**

Motorcycle traffic fatalities, although having a decreasing trend during the last decade with a better slope than most other traffic injuries, remain to be a major public health in north-west of Iran. There is a need for effective interventional programs to reduce the burden of motorcycle fatalities.

## 1. Introduction

Road traffic injuries are among the main causes of death, hospitalization, disability, and low socioeconomic status. Around 1.3 million fatal road traffic injuries and 20–50 million nonfatal injuries occur as a result of road traffic crashes each year [1, 2]. The World Health Organization states that “Without action, road traffic crashes are predicted to rise to become the 7th leading cause of death by 2030” and that about 90% of the global road traffic mortalities occur in low and middle income countries (LMICs), even though these countries have grossly half of the world's vehicles [3].

Injury epidemiology is defined as “the study of the distribution and determinants of injuries and safety-related states/events in specified populations, and the application of this study to prevent injuries and promote safety” [4]. The epidemiological aspects of injuries are not well defined in Iran and other developing countries due to loss of appropriate information systems [5]. Road traffic mortality is high in Iran compared to many countries in the world including many low and middle income countries and traffic injuries in Iran are recommended to be addressed as one of the highest public health priorities to be addressed efficiently [2, 5–7]. In most low and middle income countries, compared with high-income countries, a much higher portion of the road users include pedestrians, bicyclists, and motorcyclists, and nearly half of the fatal road traffic injuries in LMICs occur among motorcyclists. It has been shown that motorcycle injuries are a major public health problem in eastern Mediterranean region countries including Iran [8]. Despite lots of research done on road traffic safety in Iran, there is a large paucity of information based on vehicle-specific epidemiological studies, particularly in north-west of Iran. Consequently, collective evidence demonstrates vital need for mapping out the multifactorial aspects of motorcycle user fatalities. The aim of this study was to determine some epidemiological aspects of motorcycle user traffic fatalities including the crash mechanisms and injury patterns in East Azerbaijan, Iran (2006-2016).

## 2. Methods

The current cross-sectional study was conducted in East Azerbaijan province of Iran on traffic injury fatalities registered in the East Azerbaijan Forensic Medicine Organization, over a 10-year period between 21 March 2006 and 20 March 2016.

One of the 31 provinces in Iran, East Azerbaijan province, is located in north-west of the country, with coverage of approximately 47,830 km^2^, owns five percent of the whole population of the country, and reached almost 3,909,652 people, according to the recent census in 2016. The province shares borders with three countries, namely, Armenia, Republic of Azerbaijan, and Autonomous Republic of Nakhchivan, as well as three provinces, Ardabil, West Azerbaijan, and Zanjan. There are twenty districts in the province; the largest and most populated of which is Tabriz metropolitan, being the capital of East Azerbaijan with about 1,695,000 population. It is the sixth crowded capital city of the country [9].

The study's data source was the East Azerbaijan Forensic Medicine Organization covering the whole province. Under Iran's national legislation, all road traffic injury fatalities that occur within 30 days after crash are legally forced to be inspected on precise reasons for death via autopsy at the Forensic Medicine Centers. The forensic medicine centers in each district register such deaths and render all data to the lead Forensic Medicine Organization located in the capital city, Tabriz.

Totally, 9435 fatal traffic injury cases had been registered in the East Azerbaijan Forensic Medicine Organization database, for a 10-year period, from which a number of 1840 fatalities were motorcycle users.

The data collected at Forensic Medicine Organization could be categorized into following groups:

1. Crash-related data consisted of crash mechanisms, crash counterparts, inner/outer city crash, and crash year.
  - Crash mechanisms: crash of motorcycle-vehicle, vehicle-pedestrian, rollover, crash-caused fall, vehicle-fixed object, vehicle-animal, vehicle fire, others, and unknown.
  - Crash counterparts: cars, minibus, bus, pickup, truck, motorcycle, ambulance, agricultural vehicles, other vehicles, unknown, and none.
  - Types of light conditions: day light, night, twilight/dusk, and unknown.
2. Victim-related data encompasses age, sex, educational level, job, main cause of death, injured organs, and place of death.
  - Age, sex, educational level, and job.
  - Main cause of death: head trauma, bleeding, burns, asphyxia, multiple fractures, mixed causes, not defined, and others.
  - Injured organs: head and face, neck, chest and abdomen, upper limbs, vertebral column, pelvis, and lower limbs.
  - Place of death: at the crash scene, while being transported, at hospital, at home.
  - Mode of transport: ambulance, passerby vehicles, police, and unknown.
  - Role of car user: driver and passenger.

In this study, the analyzed variable of light conditions was related to the recent seven years of the study period available from September 23, 2009, to September 22, 2014.

Data analysis was carried out using Stata 13 statistical software package (Stata Corp, Texas). Descriptive statistics such as frequency, relative frequency, mean, standard deviation (SD), odds ratios (OR), and 95% confidence intervals (95% CI) were calculated. Inferential statistical methods such as Chi-squared test and multivariate logistic regression were also applied to assess potential associations between categorical scaled variables and the predictors of prehospital mortality (versus hospital mortality), respectively.

A P-value below 0.05 was considered as the statistical significance level through bivariate analysis and P < 0.1 for selecting the variables to introduce into the multivariate regression model. In order to calculate the mortality rates the provincial population was averaged between the years 2006 and 2016 to be around 3754574 people and used as the at-risk population. The study protocol was approved by the Joint Research Committee of Forensic Medicine and Road Traffic Injury Research Center, as well as the Regional Committee of Ethics in Tabriz University of Medical Sciences.

## 3. Results

A total of 9435 fatal traffic injury cases were registered in East Azerbaijan forensic medicine database through the Persian calendar years of 1385-1394 equivalent to the time period between March 2006 and March 2016. The mortality rate for all traffic fatalities occurred during the 10-year period was calculated to be 25.1 per 100000 person-years. A total of 65 (3.5%) of motorcycle user mortalities versus 1825(24%) of other traffic mortalities were females (P<0.001). Men were 8.6 times more likely to die as a motorcycle user than other road users (95% CI: 6.7-11.1). Mean age of motorcycle user victims was 32.3 years versus a mean age of 41.7 years among other traffic fatalities (P<0.001). Those in an age range of 15-30 years were 3.8 times more likely to die as a motorcycle user than other ages (95% CI: 3.4-4.2).

**Figure 1:**
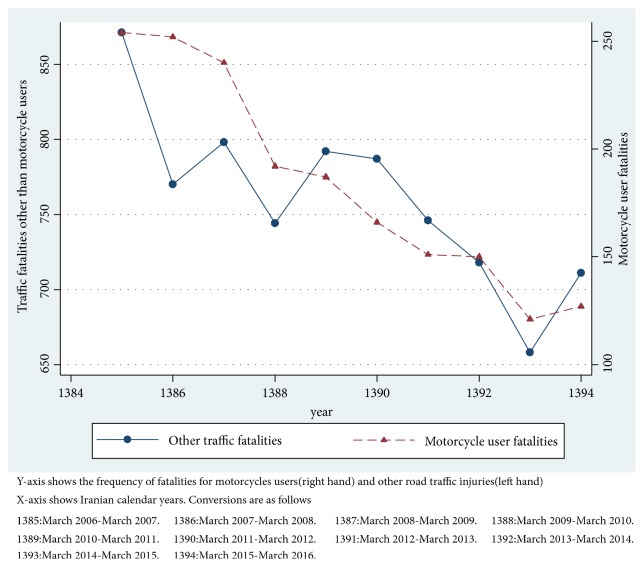
Trend of mortalities compared for motorcycle traffic injuries and other traffic injuries in East Azerbaijan Province of Iran (March 2006-March 2016).

A decreasing trend of fatal traffic accidents was observed over the study period both for the motorcycle user fatalities and other traffic injuries (Figure 1).

**Figure 2:**
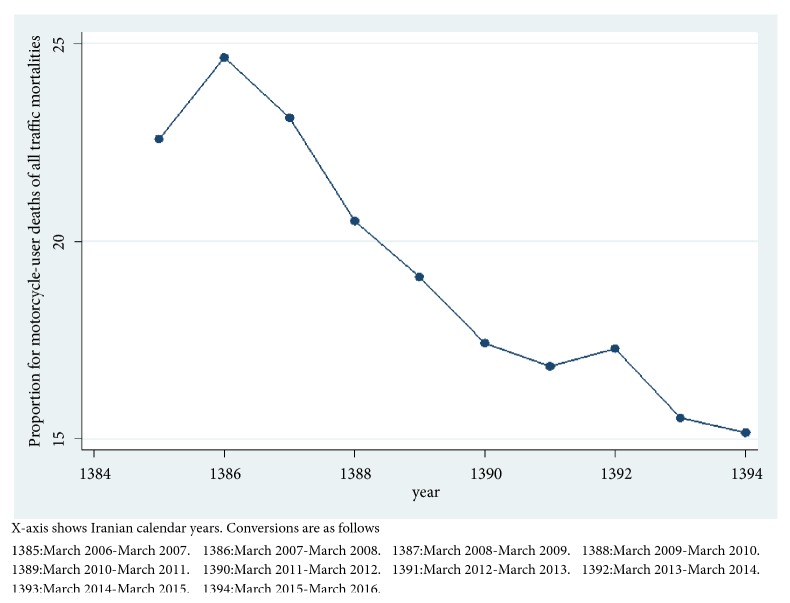
Proportions of motorcycle user mortalities on all traffic injury mortalities in East Azerbaijan Province of Iran over a 10-year period from March 2006 to March 2016.

A total of 1840(19.5%) were motorcycle users when involved in a fatal traffic accident. This figure reached 25.9% excluding the pedestrians. The mortality rate for motorcycle user traffic fatalities occurred during the 10-year period and was calculated to be 4.9 per 100000 person-years. The annual mortality rates for the last year of study were 22.3 and 3.38 per 100000, respectively, for all road traffic fatalities and motorcycle user fatalities. The percentage of motorcycle mortalities over all traffic mortalities had a decreasing trend over the past nine years from March 2007 to March 2016 reaching to a figure as low as 15.2% through the last year of study (Figure 2).

Males comprised about 96.5% (95% CI: 95.5-97.2) of the victims. Mean age of the motorcycle user victims was 32.3 years (SD: 15.2). Nearly 86% were adults aged 18 to 65 (95% CI: 84.7-87.9%), 4.6% were elderly (95% CI: 3.7-5.7), and the remainder belonged to other age groups. About 13.5% of the victims were illiterate, 56.8% had primary or guidance school education, about 5% had academic education, and the remainder had other levels of education.

About 3/4th of the victims were self-employed (40.4%), workers (18.1%), and farmers (15.2%).

Regarding the results mentioned in Table 1, the highest number of crashes occurred between vehicles and the next most frequent type of crashes was shown to be rollover of the motorcycle.

Distribution of crash counterparts among motorcycle users died due to traffic injuries is given in Table 2 showing that cars were the leading cause of mortality for motorcycle users, comprising about 34.8% of the motorcycle user mortalities. Excluding the cases when no other vehicle was engaged, cars caused 43.3% of the mortalities followed by trucks causing 16.2% of the motorcycle user deaths.

**Figure 3:**
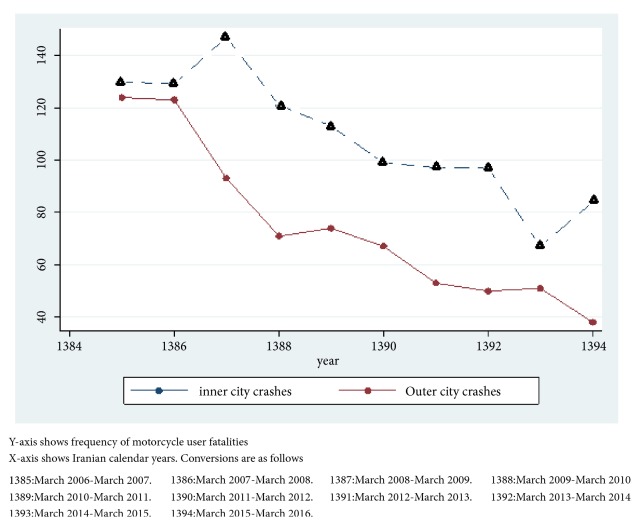
Trend of motorcycle mortalities over a 10-year period compared for inner city and outer city crashes in East Azerbaijan province of Iran.

Inner city traffic injuries were the reason for 744 (40.7%) of the motorcycle user mortalities. The decreasing pattern of motorcycle user fatalities compared with other road user fatalities over the study period is presented in Figure 3. In assessing the role of the type of counterpart vehicle on prehospital mortality, considering the other cars to be the reference group for comparison, deceased victims were 1.83 times more likely to die before getting to the hospital when the counterpart vehicle was a truck (95% CI: 1.46-2.29) followed by buses with an odds ratio of 1.66 (95 CI: 1.1-2.74).

Predictors of mortalities occurring prior to being admitted to a hospital, versus death after being admitted to hospital, were assessed. Assessing the association between accident mechanism and prehospital mortality of motorcycle-motorcycle crashes was considered as reference group. It was observed that crash with a heavy vehicle (medium and heavy trucks) increased the prehospital death likelihood by 2.5 times (95% CI: 1.7-3.8). The significant predictors of prehospital death in motorcycle traffic mortalities included age (OR=0.7, P<0.05, 95% CI:0.51-0.98), accident mechanism (OR for motorcycle-vehicle crash versus rollover=2.8, P<0.05, 95%CI: 1.1-7.1), inner city accident (OR=0.52, P<0.001, 95% CI:0.43-0.63), and type of counterpart vehicle (OR=2.8, P<0.001, 95% CI: 1.9-4.3). Head trauma was the leading cause of death accounting for 76.2% of the cases (1399 victims) followed by multiple fractures (8.1%) and mixed causes (8.06%).

Among those who died prior to hospitalization, head trauma was the main cause of death in 75.2% (653 victims) versus 77%(744 victims) among those died after hospitalization (Table 3).

Head and face were the trauma sites almost in 1609 victims of the motorcycle user mortalities (87.5%) followed by chest and abdomen in 440 victims (23.9%).

With respect to the role or position of the victim at the accident time, 1519 (82.6%) of the motorcycle user fatalities were motorcycle riders and the remainder were pillion passengers.

The information on two variables was obtained only for the last seven years of the study that were analyzed as follows.

**Figure 4:**
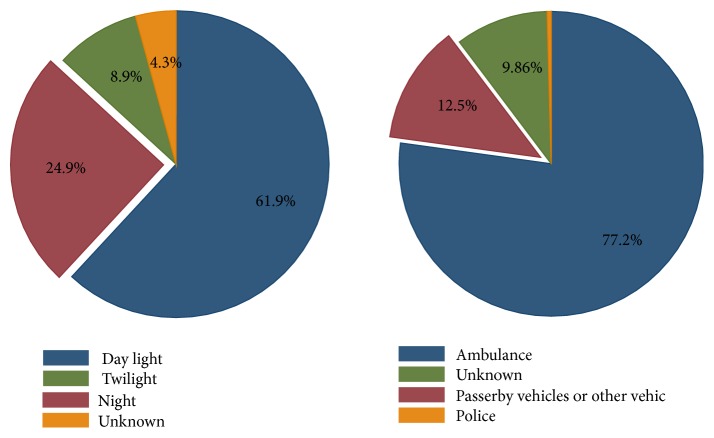
Distribution of the motorcycle user mortalities according to the light conditions of the day (left) and mode of transfer (right).

Distribution of the accidents according to the light conditions of the day as well as the mode of transferring the victim to hospital is given in Figure 4(left). Ambulance was the main vehicle for transferring the injured victims in 77.2% of the cases (Figure 4 right).

## 4. Discussion

In current study, the calculated annual road traffic death rates in East Azerbaijan were 25.1 per 100000 population and the specific mortality rate for motorcycle users was 4.9 per 100000 population. These were the average rates during the 10-year period. The annual mortality rates for the last year of study were 22.3 and 3.38 per 100000. The estimated road traffic death rate (per 100 000 population) based on the global status report on road safety 2015 was reported to be 32.1 for Iran, 21% of which belong to motorcycle users [6]. However, Iranian official statistics, although reporting mortality rates as high as 44 per 100 000 population in 2005, are indicative of lower rates compared to WHO estimates for the given year being around 23.3 deaths per 100000 population [5, 8, 10]. Our calculated mortality rate was slightly lower than the national official rates.  Table 4 provides information on road traffic fatalities and motorcycle related data. As can be found in this table motorcycle mortality proportions are not separately distinguished by some countries. Moreover, it is really hard to believe that in countries like republic of Azerbaijan only 1% of fatalities belong to motorcycles. Unfortunately, motorcycle crashes are shown to be prone to underreporting especially the single vehicle motorcycle crashes [11–13]. Although motorcycle fatalities are clearly distinguished in classified in Iranian forensic data, there is no guarantee that there is no underreporting or biased reporting for motorcycles. One concern in this regard gets back to the regulations on including the crash data only if it occurs in roads registered by ministry of road administration. Another potential field of underreporting gets back to the regulations that some mortalities could receive burial approval just by village local leaders without receiving a death certificate by an authority who is obliged to report it to the government. This, however, needs to be investigated through future research to depict the likelihood and magnitude of underreporting motorcycle injuries.

About 20% of all traffic fatalities in present study belonged to motorcycle users. The proportion decreased over the study years to as low as 15% through the last year of the study which is around the global median in 2015 in WHO global status report on road safety [6]. Even the 10-year proportion in this study is lower than the national Iranian figures in both 2013 and 2015 global status report on road safety [2, 6]. Although no specific robust study has been published to clearly define and magnify the factors and reasons for decreasing trend of traffic fatalities as a whole or specifically for motorcycle fatalities, the following general assumptions could be considered:

1. Intensified road safety legislations
2. Intensified law enforcement
3. Improved road infrastructure
4. Improved road user knowledge, attitudes, and behaviors towards traffic safety
5. Improved vehicle production safety

The mortality figures in East Azerbaijan are consistent with what World Health Organization (WHO) published about traffic deaths of car users in Iran and slightly lower than that of the Eastern Mediterranean region and some other studies [9–13], but higher than the global estimate [14], as well as some national studies [15], and in accordance with studies done in Isfahan [16], Mazandaran, Mashad, and Tehran [17–19].

Motorbikes are considered vulnerable vehicles due to their the lower safety and their low price compared to cars, which in turn makes youth who lack a riding license to be able to use them [8].

Motorcycle traffic injury victims usually have lower education and higher fatality [14, 15]. Head was the most common cause of death in present study. Head trauma is the most common cause of mortalities in most traffic injuries; however, the figure is higher for the motorcycle injuries mostly due to higher vulnerability to head trauma in motorcycle injuries especially in low and middle income countries where many riders do not wear helmets [8, 16–18]. In nonfatal motorcycle accidents, however, the extremities are also reported to be the most common part of the body involved [19–21]. Most motorcycle mortalities in present study belonged to males with a mean age of 32 years. Young men globally comprise the majority of motorcycle injuries and mortalities. In eastern Mediterranean region countries, the riders are mostly male while females are usually the pillion passengers [8]. Although motorcycle accidents crashes are more common among men, women are much more likely to die at the accident scene rather than men which may be due to the fact that women as pillion passengers are less likely to use helmets than men [22]. Moreover the pattern of energy transfer for a pillion passenger may be different from the rider or the energy tolerability among the women may be lower than men. According to a previous study in East Azerbaijan, the risk of dying after a motorcycle crash is nearly three times higher than for traffic injuries of other traffic users [23]. In present study motorcycle-vehicle crash followed by rollover comprised the crash mechanism in about 90% of the cases which is in line with most studies showing that overturning and motorcycle-vehicle crash, especially with the cars, are the two most common mechanisms of motorcycle traffic accidents. In fatal motorcycle injuries, as found in our study, the rate for vehicle-vehicle crashes is higher than nonfatal injuries showing that these types of injuries could be more fatal than other types in case of motorcycle injuries [8].

Although motorcycle mortalities showed a decreasing trend with a bigger slope than for other traffic mortalities over the past decade, even the reduced incidence in recent years is a large burden and a major public health problem needing to be adequately addressed. For any prevention work on motorcycle traffic injuries, the risk factors and target groups should be well identified and their magnitude of effect and preventability assessed. Young men and adolescents are the target groups for intensified safety promotion work. The younger people have higher risk-taking behaviors and also less likely to benefit from injuries protection measures such as having a riding license or using helmets. Psychological issues should also be seriously taken into account. The two most important psychological issues in this regard are attention deficit hyperactivity disorder and some personality disorders [24–27].

## 5. Limitations

Use of safety equipment including helmets among motorcyclists is not part of Forensic Medicine Organization registry and its association with place of death could not be addressed in current study. Moreover, no valid information to separate prehospital death into crash scenes mortality and enroute (transfer) mortalities is produced in Iranian settings. However, this is a common issue in many countries and robust evidence on separation of prehospital deaths into crash scenes and enroute mortalities is rarely available in literature.

Lack of an integrated multiorganization registry limits the opportunity to effectively link forensic data with other sources of road safety information.

## 6. Conclusion

Motorcycle traffic fatalities, although having a decreasing trend during the last decade with a better slope than most other traffic injuries, remain to be a major public health in north-west of Iran. There is a need for effective interventional programs to reduce the burden of motorcycle fatalities.

## Figures and Tables

**Table 1:** Accident mechanisms among motorcycle users died of traffic injuries in East Azerbaijan (March 2006-March 2016).

**Mechanism**	Percent	95% confidence interval	
Crash-caused Fall	1.15	0.75	1.76
Motorcycle-Vehicle Crash	77.82	75.9	79.7
Others	0.16	0.05	0.51
Rollover	11.77	10.37	13.34
Vehicle-fixed Object	8.21	7.04	9.57
Unknown	0.66	0.37	1.15

**Table 2:** Distribution of crash counterparts among motorcycle users died due to traffic injuries in East Azerbaijan (March 2006-March 2016).

**Mechanism**	**Frequency**	**Percent**	**95**%** confidence interval**	
**Agricultural vehicles**	32	1.74	1.24	2.46
**Bus**	25	1.36	0.92	2.01
**Cars**	638	34.8	32.6	37
**Minibus**	33	1.8	1.28	2.52
**Motorcycles **	185	10.08	8.78	11.55
**None**	362	19.73	18	21.6
**Other vehicles**	43	2.35	1.55	2.89
**Pickup**	232	12.6	11.2	14.25
**Truck**	238	12.97	11.51	14.59
**Unknown**	52	2.78	19.3	3.39

**Table 3:** Distribution of crash and victim related characteristics compared for place of death and as a whole among motorcycle user fatalities in East Azerbaijan, Iran.

Variables	**Pre-hospital death**		**Death after hospital ** **admission**		**Total death**	**P-value**
	Percentage (columnar)	95% CI^*∗*^ of percentage	Percentage (columnar)	95% CI of percentage		
Victim's gender						

**Male**	97	95.8-98.1	96	94.4-97	96	0.14
**Female**	2.87	1.9-4.2	4.14	3-5.6	3.54	

Victim's age group						

**<15**	3.92	2.8-5.4	2.59	1.8-3.8	3.22	0.046
**15-30**	56.91	53.6-60.2	53.16	50-56.3	54.94	
**30-45**	22.35	19.7-25.2	22.8	20.3-25.6	22.59	
**45-60**	10.37	8.5-12.6	14.3	12.2-16.7	12.44	
**>60**	6.45	5-8.3	7.15	5.7-9	6.82	

Cause of death						

**Head Trauma**	75.23	72.2-78	77.02	74.3-79.6	76.17	0.028
**Bleeding**	5.88	4.5-7.7	4.87	3.7-6.4	5.34	
**Multiple Fractures**	8.87	7.1-11	7.35	5.9-9.2	8.07	
**Mixed causes**	8.76	7-10.8	7.45	6-9.3	8.07	
**Other**	1.04	0.5-2	2.07	1.3-3.2	1.58	
**Not defined yet**	0.23	0.1-0.9	1.24	0.7-2.2	0.76	

Body organs injured						

**Head & face**	87.9	85.6-89.9	87	84.7-88.9	87.45	0.5
**Neck**	5.7	4.4-7.5	5.3	4-6.9	5.49	0.66
**Chest & abdomen**	19.5	17-22.3	27.8	25.1-30.8	23.91	<0.001
**Back spine**	2	1.2-3.1	1.4	0.09-2.4	1.68	0.4
**Upper limbs**	6	4.6-7.8	4.1	3-5.6	5	0.07
**Lower limbs**	11.3	9.3-13.5	10.2	8.5-12.3	10.76	0.49
**Pelvis**	5.9	4.5-7.6	11.3	9.4-13.4	8.7	<0.001

Counterpart crash vehicle type						

**No vehicle**	18.24	15.8-21	21.01	18.6-23.7	19.71	<0.001
**Cars**	29.1	26.2-32.2	39.86	36.8-43	34.77	
**Motorcycles**	10.28	8.4-12.5	9.94	8.2-12	10.1	
**Buses & minibuses**	3.93	2.8-5.4	2.48	1.7-3.7	3.17	
**Pickups**	13.74	11.6-16.2	11.59	9.7-13.8	12.61	
**Camionets camions or trailers**	19.28	16.8-22.1	7.35	5.9-9.2	12.99	
**Other vehicles**	5.43	4.1-7.2	7.76	6.2-9.6	6.66	

Crash location						

**Outer city**	66.71	63.5-69.8	52.56	49.4-55.7	59.29	<0.001
**Inner city**	33.29	30.2-36.5	47.44	44.3-50.6	40.71	

*∗*: confidence interval.

**Table 4:** Comparing road traffic mortality data for Iran and its land neighbors with a focus on motorcycles.

Country	Reported road traffic mortality rate in 2014	Estimated road traffic mortality rate in 2014	Percentage of deaths by motorcycle users	Rider helmet wearing rate	Helmet use enforcement score
Iran	23.2	32.1	21%	35%	5/10
Turkey	4.9	8.9	4%	DNA	3/10
Azerbaijan	12.4 (for 2012)	10 (for 2012)	1%	DNA	5/10
Iraq	17.7	20.2	DNA	DNA	2/10
Armenia	10.6	18.3	DNA	DNA	7/10
Turkmenistan	15.7	17.4	<1%	DNA	10/10
Tajikistan	5.75	18.8	DNA	DNA	6/10
Pakistan	4.1 (only crash scene)	14.2	DNA	10%	2/10
Afghanistan	4.6 (only crash scene)	15.5	DNA	DNA	DNA

Comparison data are based on global status report on road safety 2015.

DNA: data not available.

## Data Availability

Data on which this article was written could be available as innominate data upon request and approval from East Azerbaijan Province Forensic Medicine Organization and Tehran Forensic Medicine Research Center.

## Abbreviations

LMICs: Low and middle income countries
SD: Standard deviation
OR: Odds ratios
CI: Confidence interval.

## References

[1] Peden M., Scurfield R., Sleet D., Mohan D., Hyder A., Jarawan E. (2004). *World report on road traffic injury prevention*.

[2] World Health Organization (2013). *Global Status Report on Road Safety 2013 :Supporting a Decade of Action*.

[3] Fact Sheets on Road Traffic Injuries. http://www.who.int/mediacentre/factsheets/fs358/en/.

[4] Sadeghi-Bazargani H. (2012). Injury epidemiology and publishing injury research.. *Journal of Injury and Violence Research*.

[5] Sadeghi-Bazargani H., Ayubi E., Azami-Aghdash S. (2016). Epidemiological patterns of road traffic crashes during the last two decades in Iran: a review of the literature from 1996 to 2014. *Archives of Trauma Research*.

[6] World Health Organization (2015). *Global Status Report on Road Safety 2015*.

[7] Azami-Aghdash S., Abolghasem Gorji H., Shabaninejad H., Sadeghi-Bazargani H. (2017). Injury epidemiology in Iran: a systematic review. *Journal of Injury and Violence Research*.

[8] Abedi L., Sadeghi-Bazargani H. (2017). Epidemiological patterns and risk factors of motorcycle injuries in Iran and Eastern Mediterranean Region countries: a systematic review. *International Journal of Injury Control and Safety Promotion*.

[9] Tabriz District. http://ostan-as.gov.ir.

[10] Official forensic medicine statistics. http://www.lmo.ir/web_directory/53349-%D8%A7%D8%B7%EF%BB%BC%D8%B9%D8%A7%D8%AA-%D8%A2%D9%85%D8%A7%D8%B1%DB%8C.html.

[11] Amoros E., Martin J.-L., Laumon B. (2006). Under-reporting of road crash casualties in France. *Accident Analysis & Prevention*.

[12] Haworth N. How valid are motorcycle safety data.

[13] Alsop J., Langley J. (2001). Under-reporting of motor vehicle traffic crash victims in New Zealand. *Accident Analysis & Prevention*.

[14] Aghamolaei T., Tavafian S. S., Madani A. (2011). Prediction of helmet use among iranian motorcycle drivers: an application of the health belief model and the theory of planned behavior. *Traffic Injury Prevention*.

[15] Hasselberg M., Laflamme L., Ringbäck Weitoft G. (2001). Socioeconomic differences in road traffic injuries during childhood and youth: a closer look at different kinds of road user. *Journal of Epidemiology and Community Health*.

[16] Hatamabadi H., Vafaee R., Haddadi M., Abdalvand A., Esnaashari H., Soori H. (2012). Epidemiologic study of road traffic injuries by road user type characteristics and road environment in Iran: a community-based approach. *Traffic Injury Prevention*.

[17] Heydari S. T., Hoseinzadeh A., Ghaffarpasand F. (2013). Epidemiological characteristics of fatal trafficaccidents in fars province, Iran: a community-based survey. *Public Health*.

[18] Janmohammadi N., Pourhossein M., Hashemi S. R. (2009). Pattern of motorcyclist's mortality in Mazandran Province, Northern Iran. *Iranian Red Crescent Medical Journal*.

[19] Araqi E., Vahedian M. (2007). Study on susceptible and damages from motorcycle accidents in Mashhad in 2005. *Quarterly of Horizon of Medical Sciences*.

[20] Zargar M., Khaji A., Karbakhsh M. (2006). Pattern of motorcycle-related injuries in Tehran, 1999 to 2000: a study in 6 hospitals. *Eastern Mediterranean Health Journal*.

[21] Hefny A. F., Barss P., Eid H. O., Abu-Zidan F. M. (2012). Motorcycle-related injuries in the United Arab Emirates. *Accident Analysis & Prevention*.

[22] Dovom H. Z., Shafahi Y., Dovom M. Z. (2013). Fatal accident distribution by age, gender and head injury, and death probability at accident scene in Mashhad, Iran, 2006-2009. *International Journal of Injury Control and Safety Promotion*.

[23] Bazargani H. S., Vahidi R. G., Abhari A. A. (2016). Predictors of survival in motor vehicle accidents among motorcyclists, bicyclists and pedestrians in Tabriz, Iran. *Trauma Monthly*.

[24] Sadeghi-Bazargani H., Abedi L., Mahini M., Amiri S., Khorasani-Zavareh D. (2015). Adult attention-deficit hyperactivity disorder, risky behaviors, and motorcycle injuries: a case-control study. *Neuropsychiatric Disease and Treatment*.

[25] Özkan T., Lajunen T., Dogruyol B., Yildirim Z., Çoymak A. (2012). Motorcycle accidents, rider behaviour, and psychological models. *Accident Analysis & Prevention*.

[26] Haqverdi M. Q., Seyedabrishami S., Groeger J. A. (2015). Identifying psychological and socio-economic factors affecting motorcycle helmet use. *Accident Analysis & Prevention*.

[27] Sadeghi-Bazargani H., Hasanzadeh K., Salarilak S., Amiri S., Golestani M., Shahedifar N. (2018). Evaluating the relationship between adult attention-deficit/hyperactivity disorder and riding behavior of motorcyclists. *Journal of Injury and Violence Research*.

